# Gender differences in the association of perceived social support and social network with self-rated health status among older adults: a population-based study in Brazil

**DOI:** 10.1186/1471-2318-13-122

**Published:** 2013-11-15

**Authors:** Silvana C Caetano, Cosme MFP Silva, Mario V Vettore

**Affiliations:** 1 Municipal Health Secretariat of Rio de Janeiro, Rua Tiradentes, 195, 1301, Niteroi, Rio de Janeiro, RJ CEP: 24210-510, Brazil; 2 Department of Epidemiology and Quantitative Methods in Health, Sergio Arouca National School of Public Health, Oswaldo Cruz Foundation, Rua Leopoldo Bulhões, 1480, Rio de Janeiro, RJ CEP: 21041-210, Brazil; 3 Unit of Dental Public Health, School of Clinical Dentistry, University of Sheffield, 19 Claremont Crescent, Sheffield S10 2TA, UK

**Keywords:** Older adults, Perceived social support, Social networks, Social inequality

## Abstract

**Background:**

Older adults are more likely to live alone, because they may have been predeceased by their spouse and friends. Social interaction could also be reduced in this age group due by limited mobility caused by chronic conditions. Therefore, aging is frequently accompanied by reduced social support, which might affect health status. Little is known about the role of gender in the relationship between social support and health in older adults. Hence, the present study tests the hypothesis that gender differences exist in the relationship between perceived social support, social network, and self-rated health (SRH) among older adults.

**Methods:**

A cross-sectional study using two-stage probabilistic sampling recruited 3,649 individuals aged 60 years and above. Data were collected during the national influenza vaccination campaign in Rio de Janeiro, Brazil, in 2006. Individual interviews collected information on SRH, perceived social support, social network, and other covariates. Multivariate logistic regression analyses using nested models were conducted separately for males and females. Independent variables were organised into six blocks: (1) perceived social support and social network, (2) age group, (3) socioeconomic characteristics, (4) health-related behaviours, (5) use of health care services, (6) functional status measures and somatic health problems.

**Results:**

Older men who did not participate in group activities were more likely to report poor SRH compared to those who did, (OR = 1.63; 95% CI = 1.16–2.30). Low perceived social support predicted the probability of poor SRH in women (OR = 1.64; 95% CI = 1.16–2.34). Poor SRH was associated with low age, low income, not working, poor functional capacity, and depression in both men and women. More somatic health problems were associated with poor SRH in women.

**Conclusions:**

The association between social interactions and SRH varies between genders. Low social network involvement is associated with poor SRH in older men, whereas low perceived social support is associated with poor SRH in older women. The hypothesis that the relationship of perceived social support and social networks to SRH differs according to gender has been confirmed.

## Background

Self-rated health (SRH) is considered to be a valid, reliable, and robust measure of health status as well as a predictor of mortality among older people [1,2]. A number of studies have shown that SRH is closely linked with social support and social network [3-8].

### Social support and social network and SRH

Social support and social networks are interconnected terms; the former is broadly defined as the availability of interpersonal relationships and supportive persons while the latter involves the web of social relationships including friends, family, neighbours and other connections in the social environment. Social networks are the structure through which social support is provided [9]. Perceived social support is commonly considered a form of assistance that people feel is available if needed and has been defined as information leading to the belief that one is cared for or loved, is esteemed and valued, and belongs to a social network of communication and mutual obligation [10]. Social support may be emotional (expressions of positive affect, understanding, and feelings of confidence), informational (availability of people to obtain advice or guidance), tangible/material (provision of material aid), positive social interaction (availability of other persons to have fun or relax), or affectionate (physical expressions of love and affection) [11]. The different types are embedded within an individual’s social networks, which are sources of mutual social support [12].

More recently, social relationships have been shown to have health benefits for older adults, who are more prone to social isolation due to limited access to transport, reduced contact with friends and family, and living alone [13]. Reduced social resources affect the physical and mental health of the elderly [14,15]. Low levels of social support and reduced social networks can increase the risk of morbidity, sleep problems, functional decline, and mortality [9,16-18]. Perceived social support influences engagement in leisure-time physical activities [19]. In addition, older adults who participate in community activities are more likely to be physically active [20]. Those receiving more visits from their children or relatives in the previous month were less prone to binge drinking in one study [21]. Associations between low social interaction and poor perceived health, including health-related quality of life and self-rated health (SRH), have also been demonstrated [3-8,22-24]. A recent study involving 139 countries found variation in the association between social support, volunteering, and SRH across countries, but the link between social capital and SRH occurred across low and high-income countries [7].

### Gender, social connectedness, and health

Gender differences have been found in social support and social networks across the aging process [25,26]. Social connectedness varies more by gender than any other demographic characteristic [27]. In general, women have larger and more varied social networks with more friends and more social support than men [25,26]. Men tend to maintain intimate relationships with only a few people, while women identify more people as being important to them or as people they care about [28].

Despite evidence supporting the association between social relationships, resources derived from these social relationships, and health outcomes, it is uncertain whether these associations are consistent within population subgroups, such as in men or women [8,15,20,29,30]. For example, an association between participation in community activities and physical activity was observed in elderly women but not in men [20]. In most studies, gender has been considered as a confounder and measures have been taken to control for its effect in the association between social relationship and health. Consequently, the role of gender has not been clarified. Such gender differences in social interaction and interpersonal connections may implicate gender as a determinant of health in the elderly [3,29].

### Conceptual model of the relationship between social connectedness and SRH

**Figure 1:**
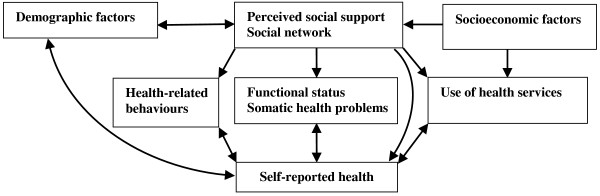
Conceptual model of the hypothesised relationships between perceived social support, social networks, other independent variables, and self-rated health in older adults.

The relationships between perceived social support, social network size, other independent variables, and SRH are summarized in Figure 1. Perceived social support and social network have direct and indirect effects on SRH [31,32]. Demographic factors such as age and sex may mediated by social support and social network and SRH since demographic factors affect social relationships and SRH. Perceived social support and social network may also be mediated by use of health services, functional status, somatic health problems and health-related behaviours [32,33]. Low social support and reduced social network result in decreased use of health services, poor functional capacity, more somatic health problems and unhealthy behaviours. We expect use of health services, functional status, somatic health problems and health-related behaviours moderate the effect of social support and social network on SRH, which means the strength of the relationship between social connectedness and SRH is dependent on these characteristics. In addition, low socioeconomic factors are associated with low use of health services and poor social relationships.

Population aging is evident in the demographic and epidemiological profiles of most countries in recent decades. Consequently, the impact of perceived social support, social network and gender requires elucidation. Therefore, the present study investigates whether gender differences exist in the effect of perceived social support and social network size on SRH in older adults.

## Methods

### Sample design and data collection

A cross-sectional study, the 1st Survey of Health Status and Life Conditions of the Elderly (*1ª Pesquisa sobre Condições de Saúde e Vida de Idosos*), was conducted in Rio de Janeiro during the Brazilian Health Care influenza vaccination campaign for older adults in 2006 [34]. This national program offers free immunisation for people aged 60 and above via public health clinics. In 2006, the campaign served approximately 77% of the Rio de Janeiro population in this age group.

Data were collected at the vaccination locations during 5 days of the campaign between April and May 2006. A total of 4,003 participants aged 60 and above were recruited. Data obtained consisted of socioeconomic characteristics, level of functioning, health-related behaviours, use of health services, presence of somatic health problems, and SRH status.

Inclusion was restricted to adult residents of Rio de Janeiro aged 60 and above who had participated in the 2006 influenza vaccination campaign and who were competent to answer the interview questions.

The sample was recruited in two stages to ensure representativeness with respect to the 10 Administrative Areas of Health Planning of Rio de Janeiro; these were considered the primary units of selection (PUS). In the first stage, a systematic sample of 60 vaccination posts from 49 health care units was drawn without replacement, considering the population of the corresponding PUS. In the second stage, a systematic sample of older adults was selected from the people visiting each vaccination post and approached for interview. The number of individuals recruited across each post was proportional to the frequency of vaccination given in the previous year. Further details are available elsewhere [34]. Sixty-seven older adults were selected from each vaccination post; thus, 4,003 people were recruited.

Interviews were conducted by 137 trained examiners and 37 supervisors. Examiners were college students attending health-related programmes who had received previous training for this study. Supervisors were staff members from the Department of Health of the Rio de Janeiro Council with previous experience in conducting health surveys.

### Ethical approval

This study was approved by the Research Ethics Committee of the Department of Health of the Rio de Janeiro Council, no. 19-A and by the Research Ethics Committee of the National School of Public Health, Fiocruz (protocol no. 145/10 CAAE: 0152.0.031.000-10, 08/11/2010). All participants who agreed to participate signed an informed consent form.

### Variables and covariates

A 70-item structured interview schedule questionnaire assessed participants’ perceived social support, social network, SRH, and other covariates.

SRH was assessed using the question: ‘Compared to other people of your age, how do you consider your health?’ Responses were made on a 5-point scale. The available range of responses was *very good*; *good, regular, poor*, and *very poor*.

Perceived social support focused on the structure of interpersonal relationships and the functional components of social support [11]. Structure of perceived social support refers to the existence of social relationships (e.g. marital status) and is most frequently measured in terms of the existence of or contact with potentially supportive persons [11]. This was assessed through the following question: ‘With whom do you live? (categories: a) alone/b) with partner or family)’. Functional social support refers to the degree to which interpersonal relationships serve particular functions. This is the perceived availability of instrumental social support from any formal or informal relationships [11]. The question assessing functional social support was ‘Are there any people you can count on or whom you can ask for help? (categories: a) Yes/b) No)’.

Social networks are the ‘web’ of social relationships surrounding the individual, consisting of the groups of people the individual is in contact with along with their characteristics and the different forms of social participation he or she engages in [35]. The following two questions were used to assess the extent of social network: ‘How often did you receive visits or visit someone else? (categories: a) None in the last 30 days/b) Once a month/c) Once every 15 days/d) One to three times a week/e) Almost every day)’ and ‘Did you attend any group activities such as religious groups, community associations, clubs, or games with friends, relatives, or acquaintances in the last 30 days? (categories: a) Yes/b) No)’. The items evaluating perceived social support and social network were adapted from previous studies in Brazil [36,37].

The demographic and socioeconomic variables were age, gender, employment status (working/not working), and years of schooling (classified into four groups: no formal education/1–4 years/5–8 years/9 years or more). Income from pension or paid work was classified into four groups, where 1 represents the minimum wage (response options: 0.0–1.0/1.1–3.0/3.1–5.0/5.1 and above) [38]. Information about kinship between participants was not collected. However, since they were systematically drawn from different vaccination posts, they were assumed to be unrelated and not residents of the same household.

Health-related behaviours referred to engaging in physical activity 5 or more times per week for at least 30 min (response options: Yes/No) [39] and smoking status (response options: current smoker/former smoker/non-smoker).

Whilst universality, comprehensive care and equity are the core principles of the national health care system in Brazil (SUS), profound inequalities in access persist and elderly people with health insurance are more likely to receive care [40]. Use of health services was assessed whether the participant had health insurance (Yes/No) and type of health services used when receiving treatment (Public/Private).

Participants’ functional status was assessed by their capacity to perform tasks in scales assessing basic and instrumental activities of daily living (ADL and IADL). ADL refers to ability to bathe, dress, use the toilet, transferring, continence, and eating. IADL gauges performance in unassisted meal preparation, housekeeping, laundering, medication management, and use of the telephone. Non-domestic activities considered in IADL are unassisted shopping for food, clothing, and medicine, and attending medical appointments and social and religious events without help. All the ADL and IADL items were scored for each activity the participant could perform independently [41]. Participants were categorized as independent (ADL and IADL = 0), partially dependent (ADL = 0 and IADL ≥ 1), and dependent (ADL ≥ 1).

Somatic health problems (joint disease, such as arthritis, arthrosis, or rheumatism; depression; hypertension; and diabetes) were measured by the following question: ‘Have you ever been diagnosed with […] by a physician or health care professional?’ (Yes/No). Participants were categorised by the number of health problems reported (0, 1, 2–4, and ≥5).

### Statistical analysis

The outcome variable was dichotomised into ‘good SRH’ (very good and good) and ‘poor SRH’ (regular, poor, and very poor) [7]. SRH status, perceived social support, social network, and other covariates were compared against gender by using Pearson’s chi-square test.

Proportions and 95% confidence intervals (95% CIs) of perceived social support, social network, and covariates were estimated for ‘good SRH’ and ‘poor SRH’ groups and stratified by gender. Comparisons of the independent variables between SRH groups were also conducted through Pearson’s chi-square test. All independent variables that had a *p* value below 0.20 in the bivariate analysis were selected for multivariate analysis. The variable ‘With whom do you live?’, used to measure perceived social support, was excluded in the multivariate analysis because of this criterion.

Multivariate logistic regression using nested models tested the association of perceived social support and social networks with SRH while adjusting for covariates. The analysis was conducted separately for each gender. Odds ratios (ORs) and 95% CIs were estimated. Stepwise forward selection of variables in different blocks was performed according to the theoretical framework (Figure 1). This approach is recommended when testing the effect of a postulated risk factor on an outcome derived from a conceptual framework while describing the hierarchical relationships between risk factors [42]. Independent variables were organised into six blocks: (1) perceived social support and social network, (2) age group, (3) socioeconomic characteristics, (4) health-related behaviours, (5) use of health care services, (6) functional status measures and somatic health problems. The significance of additional variables was tested at each stage and non-significant variables (*p* > 0.05) were excluded to reduce discrepancy between the data and the model and to obtain an model with relatively few parameters [43]. Age group and education (years of schooling) were maintained in all models as important predictors of SRH. Variance inflation factor analysis did not indicate multicollinearity between perceived social support, size of social network, and covariates.

Sample weights were used to adjust for sampling complexity. Weighted data were obtained by using a complex sample plan and were submitted to the complex samples analysis in SPSS version 17 for Windows (SPSS Inc., Chicago, IL).

## Results

Of the 4,003 individuals who agreed to participate, 354 did not meet the inclusion criteria defined, thus the final sample comprised 3,649 older adults, 22.9% of whom had poor SRH. Most were female (65.2%) and almost half were between 60 and 69 years old (Table 1). Sociodemographic characteristics, physical activity level, smoking, health insurance, somatic health problems (except diabetes), perceived social support, and participation in social groups significantly differed between men and women. Women were older and less educated, and reported lower income than men in the sample. Men scored higher on regular physical activity and tobacco use. Seventy-eight per cent reported independence in daily domestic and outside activities. The prevalence of somatic health problems varied between 17.7% (diabetes) and 62.9% (hypertension). Joint diseases, depression, and hypertension, but not diabetes, were more prevalent in women than in men. Men reported more perceived social support than women. However, women reported higher levels of participation in social group activities.

**Table 1:** Characteristics (proportions) of the study sample according to gender

	**Total(*n *** **= 3,649)**	**Women(*n *** **= 2,378)**	**Men(*n *** **= 1,271)**	***p***
Self-rated health (poor)	22.9	24.6	19.9	0.004
*Sociodemographic*				
Age group (years)				0.015
60–69	49.5	47.8	52.8	
70–79	38.1	38.8	36.9	
≥ 80	12.3	13.4	10.3	
Years of schooling				< 0.001
No formal education	6.7	7.7	4.8	
1–4 years	34.1	37.4	28.0	
5–8 years	25.6	26.4	24.1	
9 years or more	33.6	28.5	43.1	
Income level†				< 0.001
Up to 1	25.9	33.5	13.3	
1.0–3.0	37.6	39.0	35.2	
3.1 and over	36.5	27.5	51.5	
Employment status (working)	25.9	19.5	37.6	< 0.001
*Health-related behaviours*				
Regular physical activities‡	35.1	33.1	38.9	0.002
Smoking				< 0.001
Former smoker	31.8	21.7	50.7	
Current smoker	8.9	7.2	12.2	
*Use of health services*				
Health insurance	45.9	48.0	42.0	< 0.001
Use of public health service	51.0	49.7	53.8	0.063
*Functional status measures*§				
Independent (ADL/IADL = 0)	78.0	76.7	80.7	0.062
Partially dependent (ADL = 0/IADL ≥ 1)	16.6	17.5	15.0	
Dependent (ADL ≥ 1)	5.4	5.9	4.4	
*Somatic health problems*				
Joint diseases	47.4	57.2	29.1	< 0.001
Depression	24.5	28.3	17.4	< 0.001
Hypertension	62.9	65.6	57.6	< 0.001
Diabetes	17.7	17.5	18.0	0.683
*Perceived social support*				
Live accompanied	76.6	71.7	85.7	< 0.001
One or more people to count on	84.8	83.8	86.6	< 0.001
*Social networks*				
Participation in social group activities	69.2	72.5	63.2	0.017
Received visits/have visited someone				0.528
None in the last 30 days	18.7	18.4	19.1	
Once a month	16.1	16.7	15.0	
Once every 15 days	14.8	15.0	14.3	
One to three times a week/almost every day	50.5	49.9	51.6	

† Income level is presented in Minimum Wage Salaries.

‡ Regular physical activities: 5 or more times per week for at least 30 min.

§ ADL: Basic Activities of Daily Living; IADL: Instrumental Activities of Daily Living.

*p-*value refers to the significance of the Pearson’s chi-square tests.

Table 2 presents the distribution of perceived social support, social network, and covariates between SRH groups according to gender. More participants reported having persons to count on in the good SRH group for both genders. The relationship between social network variables and SRH differed between men and women. Participation in group activities was more common in the good SRH group among men, whereas low frequency of visits was associated with poor SRH among women. Years of education, income, employment status, pattern of physical activities, health care services, functional status, and somatic health problems were similar across SRH groups in both genders. Individuals with poor SRH had worse socioeconomic conditions, worse health-related behaviours, and more somatic health problems than did those with good SRH.

**Table 2:** Frequencies and 95% confidence intervals of perceived social support, social network and covariates between self-rated health groups according to gender

	**Females**			**Males**		
	**Good SRH**	**Poor SRH**	**p**	**Good SRH**	**Poor SRH**	**p**
***1st Block - Social support and social network***						
*Perceived social support*						
Live accompanied	71.2	73.3	0.304	86.4	83.6	0.254
One or more people to count on	86.2	76.1	<0.001	87.9	81.4	0.007
*Social network*						
Participation in group activities	73.2	70.6	0.192	65.1	55.0	0.002
Received visits/have visited someone			0.011			0.208
None in the last 30 days	17.0	22.9		18.8	19.9	
Once a month	16.4	17.8		14.0	19.6	
Once every 15 days	14.8	15.5		14.8	12.0	
1 to 3 times a week/almost every day	51.8	43.8		52.4	48.5	
***2nd Block - Demographic***						
Age group (years)			0.525			0.464
60 - 69	47.6	49.9		53.3	52.1	
70 - 79	39.5	37.1		37.2	36.1	
≥ 80	12.9	13.0		9.4	11.8	
***3rd Block - Socioeconomic characteristics***						
Years of schooling			<0.001			<0.001
No formal education	7.6	7.9		4.1	7.3	
1 to 4 years	35.4	44.0		25.9	36.1	
5 to 8 years	26.3	26.8		23.8	25.7	
9 years or more	30.7	21.3		46.2	31.0	
Income level†			<0.001			<0.001
Up to 1	31.1	41.4		10.9	22.4	
1.0 to 3.0	38.1	40.7		16.8	18.8	
3.1 and over	30.8	17.9		29.3	27.9	
Employment status (working)	21.4	13.7	<0.001	40.1	28.6	<0.001
***4th Block - Health-related behaviours***						
Regular physical activities‡	36.1	23.8	<0.001	42.4	25.5	<0.001
Smoking			0.889			0.123
Former smoker	21.9	21.2		50.1	52.8	
Current smoker	7.3	6.9		11.5	15.1	
***5th Block – Use of health services***						
Health insurance	51.2	37.6	<0.001	45.2	29.8	<0.001
Use of public health services	46.4	59.8	<0.001	51.0	64.3	<0.001
***6th Block - Functional status and somatic health problems***						
*Functional status measures*§			<0.001			<0.001
Independent (ADL/IADL = 0)	81.2	62.9		83.8	67.8	
Partially dependent (ADL = 0/IADL ≥ 1)	14.5	26.2		13.4	20.8	
Dependent (ADL ≥ 1)	4.3	10.9		2.8	11.4	
***Somatic health problems***						
Number of somatic health problems			<0.001			<0.001
0	7.6	0.9		17.0	4.8	
1	17.2	8.0		26.9	15.3	
2 - 4	60.3	57.6		49.9	60.5	
≥ 5	15.0	33.5		6.2	19.3	
Joint diseases	52.8	70.8	<0.001	26.8	38.7	<0.001
Depression	23.9	42.1	<0.001	13.9	31.4	<0.001
Hypertension	61.6	77.5	<0.001	54.8	68.0	<0.001
Diabetes	15.2	24.4	<0.001	16.3	25.1	0.003

† Income Level is presented in Minimum Wage Salaries.

‡ Regular physical activities: 5 or more times per week for at least 30 minutes.

§ ADL: Basic Activities of Daily Living; IADL: Instrumental Activities of Daily Living.

Logistic nested models investigated the association of perceived social support and social networks with poor SRH in women (Table 3). In Model 1, low perceived social support (people to count on) (OR 1.85; 95% CI = 1.46–2.33) and no visitors in the last 30 days (low social networks) (OR 1.40; 95% CI = 1.01–1.94) predicted poor SRH in women. Low perceived social support was associated with poor SRH after incremental adjustment for age (Model 2), socioeconomic variables (Model 3) and health-related behaviours (Model 4). Poor SRH was associated with low perceived social support after further adjustments for use of health care services, functional status and somatic health problems. In the final model (Model 6), women with low perceived social support continued to have higher probability of poor SRH (OR = 1.64; 95% CI = 1.16–2.34). Other characteristics associated with poor SRH in women were low age, low income, lack of current employment, low independence in daily activities, greater number of somatic health problems, and depression.

**Table 3:** Multivariate logistic regression of the association between perceived social support and social networks with poor self-rated health in women, controlling for covariates

	**Model 1**	**Model 2**	**Model 3**	**Model 4**	**Model 5**	**Model 6**
	**OR 95% CI**	**OR 95% CI**	**OR 95% CI**	**OR 95% CI**	**OR 95% CI**	**OR 95% CI**
***1st Block - Social support and social network***						
*Perceived social support*						
People to count on						
Yes	1	1	1	1	1	1
No	1.85 (1.46 – 2.33)*	1.74 (1.38 – 2.19)†	1.68 (1.20 - 2.34)†	1.65 (1.19 - 2.30)†	1.69 (1.23 - 2.34)†	1.64 (1.16 - 2.34)*
*Social networks*						
Participation in group activities						
Yes	1	1	1	1	1	1
No	1.10 (0.89 – 1.37)	1.15 (0.94 – 1.41)	1.17 (0.89 – 1.55)	1.13 (0.86 – 1.48)	1.10 (0.83 – 1.45)	1.04 (0.77 – 1.39)
Received visits/have visited someone						
1 to 3 times a week/almost every day	1	1	1	1	1	1
Once every 15 days	1.18 (0.84 – 1.66)	1.27 (0.96 – 1.68)	1.21 (0.84 – 1.73)	1.23 (0.86 – 1.76)	1.28 (0.87 – 1.87)	1.22 (0.82 – 1.82)
Once a month	1.15 (0.76 – 1.73)	1.21 (0.93 – 1.58)	0.98 (0.69 – 1.39)	0.98 (0.68 – 1.43)	0.95 (0.65 – 1.41)	1.01 (0.68 – 1.50)
None in the last 30 days	1.40 (1.01 – 1.94)*	1.46 (1.06 – 2.02)*	1.35 (0.86 – 2.12)	1.33 (0.84 – 2.11)	1.24 (0.76 – 2.02)	1.38 (0.83 – 2.29)
***2nd Block - Demographic***						
Age group (years)						
60 - 69		1	1	1	1	1
70 - 79		0.93 (0.78 – 1.11)	0.87 (0.67 – 1.14)	0.86 (0.66 – 1.13)	0.92 (0.69 – 1.22)	0.73 (0.54 – 0.99)*
≥ 80		1.08 (0.81 – 1.44)	0.99 (0.66 – 1.47)	0.95 (0.64 – 1.42)	0.89 (0.57 – 1.38)	0.82 (0.54 – 1.24)
***3rd Block - Socioeconomic characteristics***						
Years of schooling						
9 years or more			1	1	1	1
5-8 years			0.93 (0.64 – 1.36)	0.90 (0.61 – 1.31)	0.85 (0.56 – 1.28)	0.80 (0.53 – 1.20)
1-4 years			1.11 (0.78 – 1.57)	1.08 (0.76 – 1.53)	1.08 (0.74 – 1.58)	0.90 (0.61 – 1.33)
No formal education			0.70 (0.39 – 1.28)	0.65 (0.35 – 1.20)	0.57 (0.31 – 1.05)	0.52 (0.23 – 1.17)
Income level†						
3.1 and over 1			1	1	1	1
1.0-3.0			1.92 (1.31 – 2.81)†	1.89 (1.29 – 2.78)†	1.91 (1.22 – 2.99)†	2.06 (1.32 – 3.19)*
Up to 1.0			2.20 (1.49 – 3.26)†	2.09 (1.43 – 3.07)†	2.07 (1.35 – 3.17)†	2.32 (1.45 – 3.73)*
Employment status (working)						
Yes			1	1	1	1
No			2.00 (1.45 - 2.76)†	2.03 (1.46 - 2.83)†	2.02 (1.41 – 2.87)†	1.77 (1.21 - 2.59)*
***4th Block - Health-related behaviours***						
Regular physical activities‡						
Yes				1	1	1
No				1.41 (1.06 - 1.87)*	1.37 (1.01 - 1.85)*	1.21 (0.90 - 1.65)
***5th Block - Use of health services***						
Health insurance						
Yes					1	
No					1.25 (0.84 – 1.87)	
Use of health services						
Private					1	
Public					0.89 (0.59 – 1.36)	
***6th Block - Functional status and somatic health problems***						
*Functional status measures*§						
Independent (ADL/IADL = 0)						1
Partially dependent (ADL = 0/IADL ≥ 1)						1.83 (1.28 – 2.62)*
Dependent (ADL ≥ 1)						2.62 (1.49 – 4.62)*
***Somatic health problems***						
Number of somatic health problems						
0						1
1						1.76 (0.55 – 5.62)
2 - 4						2.75 (0.93 – 8.08)
≥ 5						4.00 (1.13 – 14.11)*
Joint diseases						1.28 (0.96 – 1.70)
Depression						1.79 (1.29 - 2.46)*
Hypertension						1.39 (0.99 – 1.95)
Diabetes						1.13 (0.77 – 1.66)

† Income Level is presented in Minimum Wage Salaries.

‡ Regular physical activities: 5 or more times per week for at least 30 minutes.

§ADL: Basic Activities of Daily Living; IADL: Instrumental Activities of Daily Living.

* p < 0.05; † p < 0.001.

Table 4 summaries the same analyses in men. Men with low perceived social support (OR 1.56; 95% CI = 1.07–2.28) and small social networks (no participation in group activities) (OR 1.54; 95% CI = 1.17–2.02) were more likely to report poor SRH (Model 1). Lack of participation in group activities (low social network) continued to be associated with poor SRH after adjusting for covariates (Model 2 to Model 6). In the fully adjusted model (Model 6), men who did not participate in group activities were 1.63 times more likely to report poor SRH than those who participated (95% CI = 1.16–2.30). Poor SRH was also associated with low age, low income, lack of current employment, low independence in daily activities, and depression (Table 4).

**Table 4:** Multivariate logistic regression of the association of perceived social support and social networks with poor self-rated health in men, controlling for covariates

	**Model 1**	**Model 2**	**Model 3**	**Model 4**	**Model 5**	**Model 6**
	**OR 95% CI**	**OR 95% CI**	**OR 95% CI**	**OR 95% CI**	**OR 95% CI**	**OR 95% CI**
***1st Block - Social support and social network***						
*Perceived social support*						
People to count on						
Yes	1	1	1	1	1	1
No	1.56 (1.07 – 2.28)*	1.58 (1.08 – 2.31)*	1.28 (0.74 – 2.20)	1.32 (0.77 - 2.29)	1.43 (0.75 - 2.72)	1.10 (0.62 – 1.94)
*Social networks*						
Participation in group activities						
Yes	1	1				
No	1.54 (1.17 – 2.02)*	1.52 (1.15 – 2.01)*	1.69 (1.24 – 2.30)*	1.57 (1.13 – 2.18)*	1.63 (1.17 – 2.26)*	1.63 (1.16 – 2.30)*
***2nd Block - Demographic***						
Age group (years)						
60 - 69		1	1	1	1	1
70 - 79		0.93 (0.72 – 1.21)	0.65 (0.46 – 0.92)*	0.64 (0.44 – 0.93)*	0.73 (0.49 – 1.08)	0.49 (0.34 – 0.73)*
≥ 80		1.17 (0.75 – 1.81)	0.90 (0.49 – 1.63)	0.92 (0.51 – 1.67)	0.98 (0.52 – 1.82)	0.71 (0.34 – 1.47)
***3rd Block - Socioeconomic characteristics***						
Years of schooling						
9 years or more			1	1	1	1
5-8 years			1.43 (0.89 – 2.30)	1.41 (0.87 – 2.27)	1.42 (0.86 – 2.34)	1.26 (0.76 – 2.11)
1-4 years			1.60 (0.99 – 2.57)	1.59 (0.97 – 2.60)	1.79 (1.08 – 2.98)*	1.44 (0.83 – 2.48)
No formal education			2.06 (0.99 – 4.31)	1.95 (0.91 – 4.17)	2.09 (0.87 – 5.02)	1.24 (0.46 – 3.34)
Income level†						
3.1 and over 1			1	1	1	1
1.0-3.0			1.18 (0.78 – 1.81)	1.15 (0.75 – 1.76)	1.06 (0.68 – 1.66)	1.18 (0.71 – 1.96)
Up to			2.05 (1.27 - 3.31)*	1.86 (1.12 - 3.08)*	2.03 (1.16 – 3.55)*	1.87 (1.07 – 3.26)*
Employment status (working)						
Yes			1	1	1	1
No			1.67 (1.20 - 2.32)*	1.63 (1.15 - 2.30)*	1.63 (1.14 - 2.33)*	1.47 (1.01 – 2.13)*
***4th Block - Health-related behaviours***						
Regular physical activities‡						
Yes				1	1	1
No				1.57 (1.03 – 2.38)*	1.59 (1.01 – 2.51)*	1.42 (0.91 – 2.22)
Smoking						
Non-smoker				1		
Former smoker				1.24 (0.85 – 1.79)		
Current smoker				1.45 (0.80 – 2.61)		
***5th Block - Use of health services***						
Health insurance					1	
Yes					1.26 (0.70 – 2.26)	
No						
Use of health services						
Private					1	
Public					0.86 (0.45 - 1.64)	
***6th Block - Functional status and somatic health problems***						
*Functional status measures*§						
Independent (ADL/IADL = 0)						1
Partially dependent (ADL = 0/IADL ≥ 1)						1.45 (0.93 – 2.28)
Dependent (ADL ≥ 1)						3.44 (1.70 - 6.97)*
***Somatic health problems***						
Number of somatic health problems						
0						1
1						1.57 (0.70 – 3.49)
2 - 4						2.41 (0.96 – 6.09)
≥ 5						3.97 (0.98 – 16.00)
Joint diseases						1.03 (0.69 - 1.53)
Depression						2.04 (1.26 - 3.31)*
Hypertension						1.07 (0.65 - 1.76)
Diabetes						1.34 (0.78 - 2.34)

† Income Level is presented in Minimum Wage Salaries.

‡ Regular physical activities: 5 or more times per week for at least 30 minutes.

§ADL: Basic Activities of Daily Living; IADL: Instrumental Activities of Daily Living.

* p < 0.05; † p < 0.001.

## Discussion

In this present study, low perceived social support and a small social network was associated with poor SRH in older adults. Social connectedness differed between elderly men and women and its effect on SRH varied significantly. The findings confirm the hypotheses that perceived social support and social network size differ between older men and women and that the relationship between perceived social support, social network size, and SRH differs in older men and women.

Our findings are also supported by several studies highlighting the association between social connectedness and SRH [3-8,22,23]. The relationship between low levels of social support and poor SRH lend support to some studies involving adults [4] and older adults [6]. Likewise, a large-scale study involving 139 low-, middle-, support from friends and relatives in adults [7].

There are some potential mechanisms whereby health benefits can accrue from social support from relatives in the elderly population. Social, behavioural, psychosocial and physiological pathways are implicated in the effects of poor social connectedness on the health of older adults [31,32]. The structure of social networks may influence disease through social support and behavioural mechanisms. Behaviours are related to social influence, levels of social engagement and participation, contact with infectious disease and access to material goods and resources [31,32]. Such behavioural and psychosocial mechanisms may operate simultaneously and affect downstream factors via biologic and physiologic pathways. In addition, psychosocial pathways can operate through cognitive and emotional states such as self-efficacy, social integration and self-steem. Finally, poor social connectedness may directly affect health if social isolation is related to stress [6]. In older adults, social support help in coping with the stress of chronic illness or stressful life events to maintain immune function and neuroendocrine and cardiovascular activity [6,9,30,44]. However, the possible influence of social connectedness on SRH in women was not found in Finland [3] and Russia [8]. Women may not derive the same health benefits from social relationships as men. The different gender roles in the society, socializing patterns and cross-cultural variations between countries may explain such discrepancies.

Having a small social network predicted the likelihood for a poor SRH in this and other studies [3,4,7,8]. Social network size is intrinsically associated with social integration and psychological well-being. Different formal and informal social networks have been associated with poor SRH such as membership of religious groups, associations, volunteering for an organisation and voluntary working [3,5,7]. Such networks can benefit health, as they foster trust, self-esteem, and cooperation [44]. Social groups can also influence health through health-related behaviours such as physical activity, binge drinking, functional capacity, cost-related medication non-adherence, and access to and use of medical care [18-21,29,45]. However, the positive effect of social networks on health appears to be more relevant for men than women since similar gender difference was found in other studies where social network size was associated with SRH only in men [3,8]. To our knowledge, however, ours is the first study to demonstrate a gender difference of perceived social support and social network on SRH in a single study of older people.

Few studies have analysed the influence of poor social connectedness on health specifically between genders. In general, women tend to have more close relationships than men, although men usually have larger social networks [46]. Therefore, the different types of social support and networks may operate in different ways and with different impacts on health between genders. Another potential explanation is the difference in the role of social interactions in lifestyle and health-related behaviours between genders. In a recent study, older women who participated in community activities were more likely to be physically active. However, this association was not found in older men [20].

In our study, older men spent less time alone and had more people to count on (higher perceived social support), but more women participated in group activities. Such gender differences suggest that older women have larger social networks, which are usually built during adult life. However, these large social networks did not correspond to a more perceived social support. At the same time, older men had less social interaction via participation in group activities. Yet, they perceived their close social relationships as more supportive.

Close social ties and perceived social support may determine SRH in older women. On the other hand, non-participation in group activities appeared to be an important aspect of social life that negatively affected SRH in older men. For older men, unspecific and broad social connectedness as well as low social isolation had a greater impact on perceived health. It can be argued that the differences in social connectedness between older men and women could have influenced differences in perceived health status.

Early studies employing analysis stratified by gender assessed the relationship of social support and social ties with mental health and risk markers for chronic diseases [46-48]. Different types of social support acted differently as risk factors for psychological distress in men and women. Emotional support from close relations was associated with good mental health only in men. However, negative aspects of close relationships predicted poor mental health in both men and women [47]. Emotional support (from up to four close people) benefited mental health only in women. The effect of social support and network on subsequent psychological distress was similar in both genders [46]. In another study, more social ties varied inversely with allostatic load as measured by clinical and serological risk factors for chronic disease in both genders. However, high emotional support was inversely associated with allostatic load only in men [48].

In the present study, SRH was dichotomised because few participants considered their health as “poor” or “very poor” (women: 3.2%; men: 1.7%). SRH has frequently been assessed as a binary outcome in previous studies [3,7,8]. Similar findings arouse if ordinal regression was used with ordinal categories. Low perceived social support was associated with poor SRH in women using ordinal regression (adjusted OR = 1.47; 95% CI: 1.16 – 1.86). Low social network increased the likelihood of poor SRH in men when ordinal regression (adjusted OR = 1.30 95% CI: 1.21 – 2.24) was used.

The strengths of the present study were its robust sample and the theoretical model used to test the associations. In addition, the nature of the variables was accounted for in the nested modeling procedure.

However, our study has some limitations. We included only participants in the vaccination campaign who could complete the interview. Thus, the findings reflect older adults living independently or with low levels of dependency but may not be generalisable to all elderly adults. In addition, although the items used to assess social support and social network size were derived from theoretical constructs, they were not previously validated for the studied population. The cross-sectional design also restricts inferences about causal relationships.

## Conclusions

The association of social connectedness with SRH differed across genders. Low social network involvement is associated with poor SRH in older men, whereas low perceived social support is associated with poor SRH in older women. The findings of this study confirm the hypothesis that the relationship of perceived social support and social network with SRH differs between older men and women.

## Competing interests

The authors declare that they have no competing interests.

## Authors’ contributions

SC was involved in design of the study, acquisition of data, analysis and interpretation of the data, interpretation of the results, and drafting of the manuscript. CMF developed the statistical framework for data analysis, conducted the statistical analysis, interpreted the data, and reviewed the manuscript. MV was involved in the conception and design of the study, development of the statistical framework for data analysis, and drafting of the manuscript. All authors read and approved the final manuscript.

## Pre-publication history

The pre-publication history for this paper can be accessed here:

http://www.biomedcentral.com/1471-2318/13/122/prepub
